# Distinguished American Physicians Lately Deceased

**Published:** 1853-08

**Authors:** 


*Distinguished American Physicians lately deceased.*—The medical profession of this country has lately been deprived, by death, of several of the most distinguished of its senior members.

The venerable Dr. Chapman, of Philadelphia, died on the 2d ult. For nearly half a century Dr. Chapman, as a teacher, writer, and practitioner, ranked among the master minds in the profession. A professor in the time-honored University of Pennsylvania, author of several successful works, enjoying a large practice, distinguished for his wit and social disposition, no member of the profession, during this long period, was more widely known, or more universally respected. He has at length ended his earthly career as full of years, as of honor.

In the death of Dr. Wm. Beaumont, medical science has lost one of the most eminent of her votaries. Availing himself of the opportunity of examining ocularly the internal appearance of the stomach, and withdrawing its contents at will, in the remarkable case of Alexis St. Martin, his well conducted experiments and observations may be said to have formed an important epoch in physiology, with which the name of Beaumont will never cease to be associated. Dr. B. was for twenty years a member of the medical staff of the army of the United States. He resigned in 1837, and settled at St. Louis, where he soon acquired an extensive practice, which he continued to hold up to his last illness. As a citizen, and friend, he was highly esteemed by all who enjoyed the privilege of knowing him in these relations. He died at the age of 68.

The death of Dr. Charles Caldwell took place, at Louisville, Ky., on the 9th ult. Dr. Caldwell, it is stated, had "reached the advanced age of about 90 years, being one of the oldest, if not the very oldest physician in the United States."* He was endowed with a vigorous intellect, and as a medical writer and lecturer, in the days of his intellectual vigor, had few equals.

It is to be hoped that biographies of these distinguished physicians, if not seasonably prepared by those who may volunteer such a tribute to their memories as an offering of personal respect, will be entrusted to competent hands by the medical societies of the states, respectively, in which they resided. Justice alike to the dead, and the living, demands not less than this. But how often is this duty overlooked, or imperfectly performed!

* Transylvania Med. Journal.

